# Portable Measurement System for *in situ* Estimation of Oxygen and Carbon Fluxes of Submerged Plants

**DOI:** 10.3389/fpls.2021.765089

**Published:** 2021-11-05

**Authors:** Annika Schrumpf, Andreas Lengerer, Nicola Schmid, Marian Kazda

**Affiliations:** Institute for Systematic Botany and Ecology, Ulm University, Ulm, Germany

**Keywords:** carbon metabolism, *in situ* measurement, photosynthesis, optical sensors, aquatic plants

## Abstract

The metabolism of submerged plants is commonly characterized by oxygen development. The turnover rates of carbon dioxide and other inorganic carbon species, however, are assessed only at distinct points in time after incubation or calculated through shifts in pH and total alkalinity. A novel three parameter measurement system was developed in order to improve this issue and to gain a better understanding of the metabolism of aquatic plants. It allows the simultaneous and continuous assessment of oxygen concentration, partial pressure of carbon dioxide and pH with optical sensors without the need of taking water samples. Plants or plant parts can be enclosed in a chamber, while the surrounding water is either flushed through or circulated within the system. The method was evaluated in regards to measurement time and possible stress reactions during measurement. Its applicability *in situ* was confirmed with *Elodea nuttallii* and *Ceratophyllum demersum*. The measurement system will enable deeper insights into the metabolism and response of aquatic plants to changing environmental conditions, especially related to carbon fixation.

## Introduction

In the last years the awareness for aquatic habitats and aquatic macrophytes increased, especially with view to the local and global carbon balance and the capability of aquatic systems to sequester carbon (Duarte et al., 2005; Cole et al., 2007), the so called Blue Carbon.

Several reviews characterize the currently used methods for the evaluation of metabolism of single specimens, species and habitats, in laboratories and *in situ* (e.g., Silva et al., 2009; Pedersen et al., 2013). The most prominent method for the evaluation of the photosynthetic rate and health status of aquatic plants is the PAM fluorometry (Beer et al., 1998). It is widely applicable and needs no sample taking or even physical contact with the measured plant as it measures the excitation of Photosystem II. However, it cannot be used to assess the carbon turnover, neither during light nor during dark conditions. To obtain information on the metabolic characteristics of an aquatic habitat, the eddy covariance technique, adapted for use under water, can be applied (Berg et al., 2003; Attard et al., 2019). This method is promising for the observation of ecosystems, but cannot be used to evaluate the contribution of single plant specimens as it measures the oxygen fluxes of wider areas. Furthermore, up to now, the turnover of carbon species is not evaluated in this method.

Incubation chambers are used for *in situ* measurements in aquatic habitats, e.g., in benthic habitats (Silva et al., 2008; Roth et al., 2019) or for corals (Camp et al., 2015). Different sensor technologies for oxygen, light and pH can be implemented to evaluate the activities of the organisms inside the defined volume. Enclosing single specimens as by Rodgers et al. (2015) allows the examination of the specific metabolic activities. The incubation time of the plant specimen and time span between taking the samples has an impact on the reliability of the results (Olivé et al., 2016). As incubation chambers are closed systems, photosynthetic products accumulate during illumination, leading to elevated oxygen levels, carbon dioxide depletion and rising pH levels. The photosynthetic rate and carbon turnover would then be underestimated as the change in oxygen and DIC concentration would also affect other processes than photosynthesis, e.g., photorespiration. Additionally, many aquatic plants have adapted to the described stress situations by developing carbon concentrating mechanisms (Maberly and Madsen, 2002; Yin et al., 2017). The plants can concentrate carbon species internally or use bicarbonate as carbon source. This helps to avoid ratios of oxygen to carbon dioxide near the enzyme Rubisco that would favor the oxygenase activity.

Both the oxygen and the carbon turnover rates have to be studied, to precisely evaluate the metabolism of plants and aquatic habitats (Vachon et al., 2020). In many approaches only oxygen is investigated on (Pedersen et al., 2013). Measurements are mainly conducted with optical sensors (Miller and Dunton, 2007), via Winkler titration or with Clark electrodes (Silva et al., 2009). The measurement of the carbon turnover can be examined via changes in the pH, by titration of water samples taken in distinct time intervals, or plant material can be incubated with isotopes to measure turnover rates (Nielsen, 1952; Silva et al., 2009). A new lab method using infrared gas analysis compares the CO_2_ concentration of a reference sample with air that passed water with incubated organisms (LICOR 6800-18 Aquatic Chamber). This is an addition to other IRGA applications (Silva et al., 2008) and porometers (Hussner, 2009) that can be used on emerged aquatic plant parts and in incubation chambers with an integrated part for the formation of an air water equilibrium of the gas concentrations.

There is a lack of precise data on carbon uptake or release of submerged plants under differing environmental and stress conditions. In the last years optical sensor foils were developed with the possibility of measuring the pCO_2_ continuously without the need of water sample extraction. The foils were implemented on research cruises (Staudinger et al., 2018), under live haul conditions (Thomas et al., 2017) or in chambers with enclosed leaves of *Rhizophora mucronata* under aquatic conditions (Ulqodry et al., 2016). By augmenting the measurement of the pCO_2_ with the measurement of the pH, the other carbon species concentrations could be examined as well. Still, to our knowledge, such measurements were not conducted on individual aquatic specimens *in situ*.

In this paper we introduce a novel method for continuous, simultaneous assessment of the three parameters oxygen concentration, pCO_2_ and pH using optical sensors and a small incubation chamber. The handheld size allows flexible mounting to plants and easy changing between specimens with minimal impact on the measurement site. The method was evaluated in respect of the measurement time, possible stress reactions during measurement in a controlled setting and was applied *in situ* on the two submerged species *Elodea nuttallii* and *Ceratophyllum demersum*.

## Materials and Methods

**FIGURE 1:**
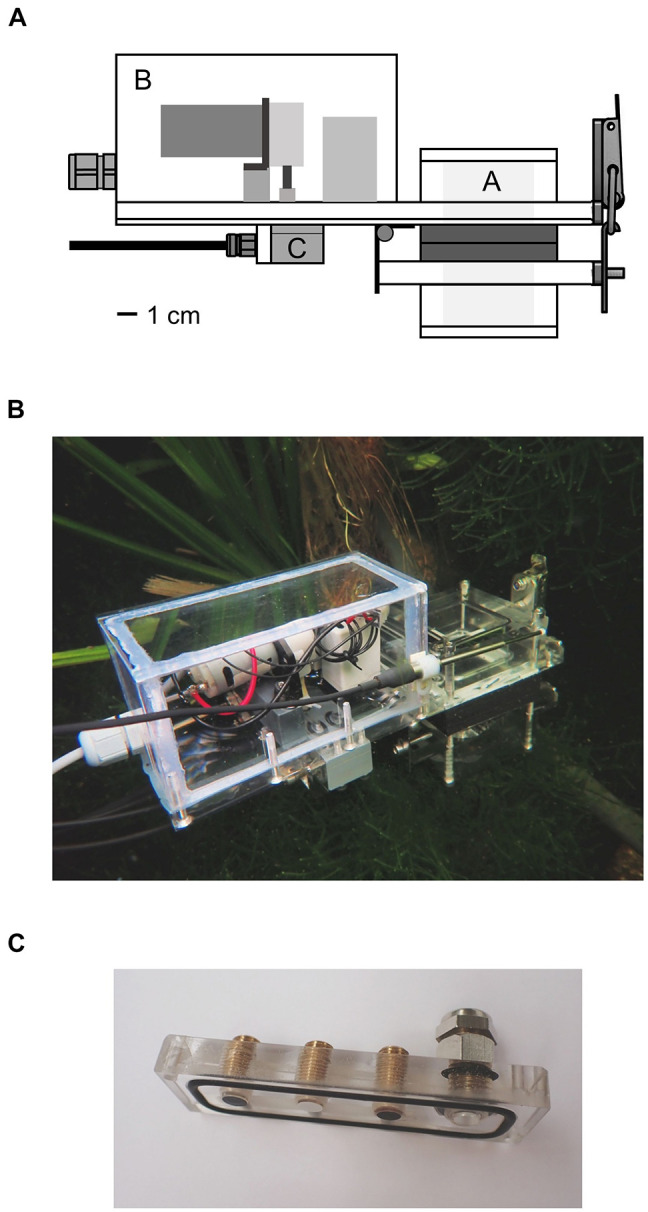
**(A)** Schema of the underwater measurement device. Plants were enclosed in the incubation chamber A by opening the lid of the chamber with the hinge. The rear part B contained a peristaltic pump and two solenoid valves which could be switched between the modes *flush* and *measurement*. Water from the surrounding was pumped through the device in the first mode and was circulated inside the device in the second mode. Optical sensor foils and a thermocouple were attached to the piece C. **(B)** Entire device in an aquarium in the Botanical Garden of Ulm University. **(C)** Sensor foils attached to the PMMA piece; fiber optics were screwed onto the piece from the other side. The thermocouple was inserted into the port on the right side.

The presented device for the *in situ* measurement of the oxygen and carbon flux of submerged plants consisted of an incubation chamber, various sensors, a pump and valves (Figure 1). The device was constructed in the workshops of Ulm University under the authors’ supervision. The salts and calibrating solutions were purchased from Merck KGaA (Darmstadt, Germany). All data analysis was performed using Excel 2016 and Python 3.7. The Python script was used to automate the calculations described in section “Data Acquisition and Processing” and to plot the Figures displayed in the Results.

### Description of the Device

The device was mainly constructed from color-less, transparent polycarbonate and had an outer size of 25 × 15 × 7 cm. The rear part contained a peristaltic pump with a flow rate of 85 ml min^–1^ and two solenoid valves (Type RVB-3R-MFEA; Takasago Electric Inc., Nagoya, Japan) which were supplied with power by an external 12 V battery pack. The power consumption of the valves and the pump was 17 W, when the pump was running with full speed. The incubation chamber and the pump and valves were connected to each other by channels of 3 mm diameter.

A plant or parts of it were inserted into the incubation chamber in the front part of the device, which was then closed tightly. A part of the incubation chamber was fixed to the device and another part functioned as lid, fastened by a hinge. This allowed the quick opening and flexible change of the included specimens. Rubber was glued to the contact surfaces of the lid to secure tight closure. The incubation chamber had a basic inner volume of 64 cm^3^ but could be extended by additional components. Including the pump, the valves and all channels, the voids of the device added up to an entire inner volume of 72 cm^3^. Conical filters with a diameter of 2.5–4.5 mm were inserted in the entry channel of the device and the exit channel of the incubation chamber and were changed regularly. The filters kept organic material, small animals, plants parts and algae from entering the device; microorganisms were not filtered out.

**FIGURE 2:**
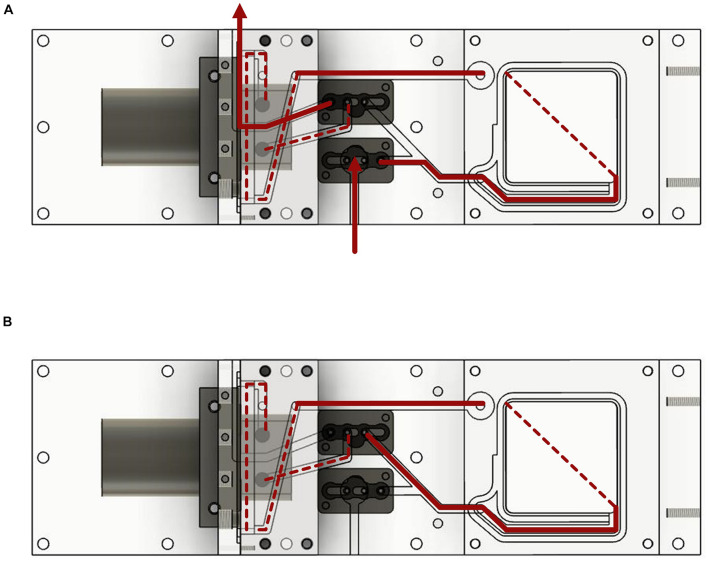
Water flow diagram of the measurement system. The device is shown from the underside. **(A)** Water flow with the valves in the open position. **(B)** Water flow with closed valves and water circulation.

Water from the surrounding was pumped into the device and flushed all voids. Depending on the position of the valves, the water then circulated through the system or entered and exited the device leading to water exchange and a rinsing process. The water in the device always passed the incubation chamber, the valves and the sensors (Figure 2). The position of the valves could be controlled in two different ways by a control unit installed between the power supply and the measurement device (the circuit diagrams can be found in Supplementary Figures 1A,B). A manual switch could be used to switch the valves between the options of measuring and flushing. In this case the pump was running the entire time. The second option was the use of an Arduino microcontroller. The duration of both valve positions could then be set, leading to a routine. As soon as the routine was started, the pump began to work.

### Sensors

The optical sensors consisted of foils with 5 mm diameter which were triggered by optical fibers (POF with SMA connector) connected to meters (SMA series; all PreSens Precision Sensing GmbH, Regensburg, Germany; characteristics can be found in the Supplementary Table 1). The meters were supplied with power by the field laptop that also collected all data via USB. For information on the operating principle and construction of optical sensors for the measurement of the oxygen concentration, partial pressure of carbon dioxide and the pH, please see the current literature (e.g., Atamanchuk et al., 2014; Fritzsche et al., 2018).

The sensor foils were attached to a sensor carrier block made of PMMA which was then screwed onto a gray, opaque PVC block on the underside of the device (Figure 1C). The sensor foils had contact to the water circulating in the device. A temperature sensor (Pt-100) was plugged into the device next to the sensor foils. The response of the luminophore coating of the sensor foils is temperature dependent (Atamanchuk et al., 2014). Therefore, the calculation of the oxygen concentration, the pH and the pCO_2_ was compensated internally in the gauges.

As the sensor foils were pre-calibrated, only those sensors were recalibrated that were re-used after a longer period of use or in storage. The oxygen sensor was recalibrated with an oxygen-free solution of Na_2_SO_3_ and with air at 100% humidity. The pH sensor was recalibrated with a one-point-calibration with a pH buffer at pH 6.8. The CO_2_ sensors were changed after longer periods of use; the time span of usage depended on the amount of conducted measurements and was between 2 and 6 months. All sensors were changed in the case of low measurement amplitudes.

The amount of PAR reaching the plant material was measured with a spherical sensor (Spherical Micro Quantum Sensor US-SQS/L with a sensor head of 3 mm diameter; Walz GmbH, Effeltrich, Germany) and logged with a LI-1500 Light Sensor Logger (LI-COR Biosciences, Lincoln, United States). The sensor attached to the side of the incubation chamber measured the incoming radiation as photosynthetic photon flux density (PFD).

### Measurements

The feasibility of the method was addressed by two experimental setups. In a first step the method was used under laboratory conditions to find out whether the plants were stressed through prolonged incubations in the chamber; the second step included testing the applicability *in situ*.

All measurements were conducted using a standardized protocol. The device was put into the water at the measurement site and was rinsed without plant material inserted. As soon as the optical sensors were equilibrated and showed constant values, a plant was enclosed in the incubation chamber and the lid was closed tightly. Measurements of the metabolic activity of the plants were performed for 20 min with circulating water (i.e., valves in closed position). Afterward, the valves were opened and the incubation chamber was rinsed with surrounding water for at least 10 min until the displayed values of the oxygen concentration, pCO_2_ and pH were constant. The plant material either remained in the incubation chamber or was replaced by a new specimen during the rinsing process.

#### Data Acquisition and Processing

The gauges stored the raw data of the oxygen concentration, the pCO_2_, the pH, the atmospheric pressure and the temperature every 10–20 s in the program PreSens Measurement Studio 2; the data could by exported as xlsx file afterward. The data from the spherical light sensor were saved on the light logger and transferred to the field computer as csv file. Additionally, the projected leaf area was obtained by scanning the leaves and processing the images with ImageJ. The leaves were either flattened out or the whorls were divided and scanned individually.

The concentration of CO_2_ was determined from the pCO_2_ by applying the temperature dependent Henry law (Sander, 2015). In a next step the bicarbonate concentration was calculated from the carbon dioxide concentration, the pH and the temperature and salinity dependent acid dissociation constant (Millero et al., 2006) with the Henderson-Hasselbalch equation.

The molar fluxes of oxygen, carbon dioxide and bicarbonate were determined by linear regression for the interval between the fifth and the tenth minute after the closure of the incubation chamber. The values were normalized to the leaf area.

#### Plant Material

One hundred and fifty specimens of *Elodea nuttallii* (Planch.) St. John, Hydrocharitaceae, were taken from the pond in the Botanical Garden of Ulm University in November 2019 (IPEN DE-0-ULM-2020-F-91). The plants were put into two dark rubber containers filled with rain water to a height of 17 cm and 2 cm of sediment from the pond. The containers stood in a greenhouse with ambient light and temperatures between 8 and 12°C. The first measurements in the laboratory were conducted after 2 weeks of acclimatization; the measurements were finished at the end of December 2019.

Further measurements were conducted *in situ* in the same pond as mentioned above on six specimens of *Ceratophyllum demersum* L., Ceratophyllaceae, (IPEN DE-0-ULM-2021-F-26) and two specimens of *E. nuttallii* in summer 2020.

#### Lab and Field Experiments

##### Effects of the incubation time

Plant specimens were either measured individually for 20 min at distinct light intensities or they were measured for about 5 h for an entire light response curve in a climate chamber. The light sources in the chamber, providing different light intensities, were high pressure mercury lamps (type HPI-T 400W; Philips, Signify Holding, Hamburg, Germany). In the second case the measurement device was rinsed every 20 min for 10 min while the light intensity increased in steps from 0, 50, 100, 150, 200, 300, 500, 700 to 900 μmol photons m^–2^ s^–1^ PFD. The light intensity reaching the measurement device was not identical to the presented values due to the scattering and absorption of light in the water body. Five replications with individual specimens were carried out at each light intensity (*n* = 45) and for the light response curves in the longtime incubation (*n* = 5), respectively.

Young, clean plants without root development or external damages were chosen for the measurements. The leaf area of the specimens was between 6.4 and 28 cm^2^ (median 13.8 cm^2^). The plants were brought into the climate chamber 1 h before the measurements began and were exposed to the pre-selected light intensity. The measurements were done in a mixture of tap water and decalcified water. The measurement device was inserted 10 cm deep into the water in a black rubber container.

##### Field application

*In situ* measurements were conducted in the pond of the Botanical Garden of Ulm University in July and August 2020. At this time of the year the dense vegetation was dominated by *Elodea canadensis* MICHX., *E. nuttallii*, and *C. demersum*.

The measurement device was fixed at 10 cm below the water surface. The measurements were performed as described with the valves closed for 20 min alternated by at least 10 min rinsing. For this study, two specimens of *E. nuttallii*, both with a leaf area of 16.3 cm^2^, were measured over a longer period between 6 a.m. and 2 p.m. and six specimens of *C. demersum* were measured individually for 20 min each. The leaf area of these specimens was between 20.5 and 55.8 cm^2^ (median 29.1 cm^2^).

## Results

**FIGURE 3:**
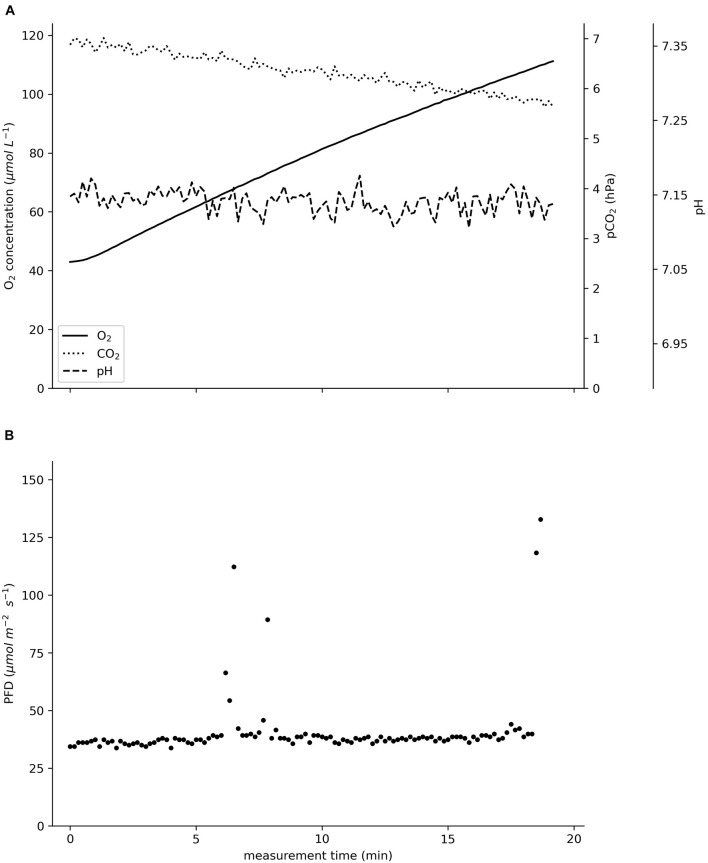
Raw data recorded *in situ* on an incubated specimen of *Ceratophyllum demersum* in August 2020 in the pond of the Botanical Garden of Ulm University. **(A)** Oxygen concentration in μmol L^–1^, pCO_2_ in hPa, pH. **(B)** Photon flux density (PFD) in μmol m^–2^ s^–1^. The total incubation time was 19 min.

The raw data in Figure 3 visualize the reciprocal changes in the oxygen concentration and pCO_2_; as the oxygen concentration rose from 43 to 111 μM, the pCO_2_ declined by 1.2 hPa from 6.9 to 5.7 hPa. The pH remained at 7.15. The sensor foils showed a high precision; especially the pCO_2_ sensor convinced with high precision at low partial pressures.

### Effects of the Incubation Time

**FIGURE 4:**
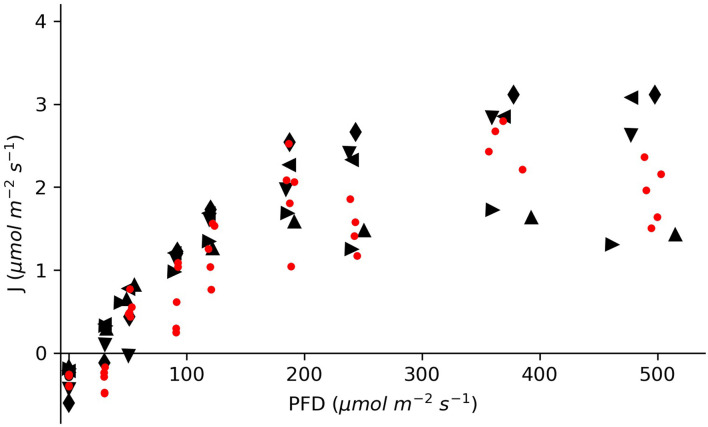
Molar oxygen flux of *Elodea nuttallii* specimens measured under differing light conditions in a climate chamber. The red dots show data for single specimens measured at distinct light intensities. The black symbols represent data for five specimens measured continuously for an entire light response curve with cycles of rinsing and measuring.

The oxygen turnover by *E. nuttallii* showed that the values of individually measured plants were in between the values of plants inserted in the chamber for more than 5 h (Figure 4). All specimens showed light saturation beginning at about 200 μmol photons m^–2^ s^–1^ and oxygen uptake in dark conditions. The maximum oxygen flux rates for the long-time measured plants were between 1.8 and 3.4 μmol O_2_ m^–2^ s^–1^.

### *In situ* Application

**FIGURE 5:**
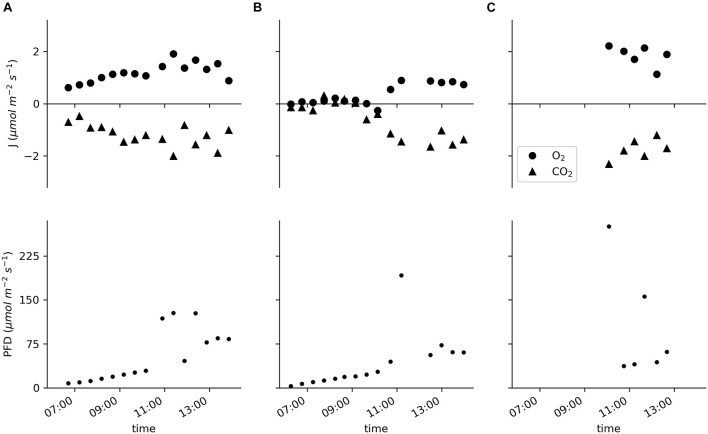
Molar fluxes of oxygen and carbon dioxide and the PFD measured *in situ* in the pond of the Botanical Garden of Ulm University. **(A,B)** Two specimens of *Elodea nuttallii* each measured for the entire displayed time period with cycles of 20 min of incubation and 10 min of water exchange. **(C)** Six specimens of *Ceratophyllum demersum* measured individually for 20 min.

Data for two specimens of *E. nuttallii* and six specimens of *C. demersum* measured *in situ* showed specific daytime-related patterns (Figure 5; the basic environmental parameters for the *in situ* measurements are listed in Supplementary Table 2). As the measurements of the *E. nuttallii* specimens were conducted from the early morning until noon, the fluxes of oxygen and carbon dioxide increased with the light intensity (Figures 5A,B).

The oxygen flux of the *E. nuttallii* specimens varied between −0.3 and 1.9 μmol m^–2^ s^–1^; the values for the oxygen flux of *C. demersum* were between 1.1 and 2.2 μmol m^–2^ s^–1^ (Figure 5C). In all cases the carbon dioxide fluxes were reciprocal to the oxygen fluxes with values between 0.1 and −2.0 μmol m^–2^ s^–1^ for *E. nuttallii* and −1.2 and −2.3 μmol m^–2^ s^–1^ for *C. demersum*, respectively.

## Discussion

### Measurement Duration

The oxygen turnover of *E. nuttallii* specimens inserted for 20 min each was compared with specimens that stayed in the device for about 5 h with periodic rinsing through opened valves. The oxygen turnover of the individually measured plants lay in between the values of the longtime incubated plants (Figure 4). Specific changes in the oxygen fluxes in this experiment would have indicated stress. As shown, a longtime incubation with periodical rinsing of the chamber does not affect the metabolic activity of the inserted plants.

The specimens used in the described experiments were originally taken from a shallow pond and then kept in rubber containers; in both sites there was no active water flow. The flow rate was set to 85 ml min^–1^ inside the incubation chamber. As the circulation was rather slow, the change from stagnant water to circulated water had no obvious effect on the turnover rate, but might have enhanced the turnover rates slightly due to thinner boundary layers (Jones et al., 2000).

The measurements in the climate chamber were conducted with an oxygen concentration of between 70 and 80% air saturation (190 and 230 μM) at the beginning. The maximum change in the oxygen concentration led to a concentration of 111% air saturation (301 μM) after 20 min of incubation at the highest possible light intensity; the absolute change was 83 μM. In the majority of the cases, the concentration did not exceed air saturation. Therefore, photorespiration should not have been enhanced in a dominant manner (Burris, 1981). By exchanging the water regularly, the depletion of carbon species and increase of oxygen were avoided, which would otherwise lead to higher oxygenase activity of Rubisco (Olivé et al., 2016). In other incubation chambers hypoxia and depletion of carbon species are avoided by enlarging the volume of the incubation chamber (Camp et al., 2015) or by shortening the incubation time (Silva et al., 2008) as conducted in this setup. When conducting measurements with a plant specimen incubated for a longer period, it is important to secure the circulation of the water through the device to reduce the effects of the accumulation of metabolic products near the leaf surface (Mass et al., 2010).

The maximum oxygen flux rates for the longtime measured plants ranged between 1.8 and 3.4 μmol O_2_ m^–2^ s^–1^. These values are in accordance with measurements on elodeids acquired from natural environments at the respective pH (Madsen et al., 1993; Nielsen, 2015), what proves the functionality of the measurement device. The rates of oxygen uptake in the dark were 5–10 times lower in comparison to oxygen release at light compensation. This ratio has been shown in other measurement setups at an oxygen concentration of around 200 μM (Simpson et al., 1980; Jones et al., 2000). Another proof is the observation of light saturation, as described by other authors for *Elodea* species (Jahnke et al., 1991; Madsen and Sand-Jensen, 1994).

### Field Application

*In situ*, the device simultaneously assessed changes in the oxygen concentration, the pCO_2_ and the pH values (Figure 3), enabling the calculation of the carbon species’ concentrations. The molar flux of carbon dioxide was reciprocal to that of oxygen (Figure 5).

The values measured for the oxygen production of *C. demersum* are in line with other measurements in the lab (Kitaya et al., 2003) and *in situ* (Goulder, 1970; Fair and Meeke, 1983). The oxygen and carbon dioxide fluxes measured on *E. nuttallii* are rather low compared to the literature (e.g., Madsen and Sand-Jensen, 1994; Nielsen, 2015) and to the experiments conducted in the laboratory in this study, mainly due to the less intense irradiation. As the light intensity was lower than 100 μmol photons m^–2^ s^–1^ during the measurements, light saturation could not be shown.

The pre-set flushing intervals of the incubation chamber with fresh water are one of the major advantages of the presented measurement device. It significantly reduces the effect of depleted or accumulated metabolic educts or products (Olivé et al., 2016). During *in situ* measurements, it also allows to keep the incubated plants in their natural diel cycle, even during longtime incubations. This represents an improvement in comparison to previously presented incubation chambers applying pumps and valves (e.g., Silva et al., 2008; Rodgers et al., 2015). When the surrounding oxygen concentration is high, e.g., in shallow ponds in the afternoon (Ganf and Horne, 1975), the incubation time could be shortened from 20 to 15 min to avoid additional oxygen accumulation.

The assessment of the pH and the partial pressure of carbon dioxide enables the calculation of the concentrations of the other inorganic carbon species, e.g., with the Henderson-Hasselbalch equation. This is particularly interesting when the examined species can use bicarbonate as carbon source, as it is the case for *E. nuttallii* and *C. demersum* (Yin et al., 2017). In this study, bicarbonate was not taken up by the incubated plants. Changes in the bicarbonate concentration could be lead back to shifts in the carbon species equilibrium as the pH and the ratio of carbon dioxide to bicarbonate stayed constant. Nevertheless, the presented method could help to understand the preferential carbon species uptake in aquatic macrophytes.

The photosynthetic quotient (PQ) is calculated as the ratio of the released oxygen to the incorporated carbon. The PQ equals 1, when photosynthesis and respiration are the only occurring processes. Several studies have shown that the PQ may differ from 1 depending on the light intensity, oxygen concentration or concentrating mechanisms (Burris, 1981; Pokorný et al., 1989; Carvalho, 2014). Furthermore, the PQ has to be calculated considering all used carbon species, i.e., carbon dioxide and bicarbonate. In various approaches to determine the metabolism of aquatic organisms or habitats, only the oxygen turnover is monitored; the carbon turnover is left out, calculated by changes in the pH or measured irregularly (Pedersen et al., 2013).

The PQ in this study was based on carbon dioxide as bicarbonate was not used. For the six *C. demersum* specimens the PQ ranged between 0.94 and 1.18 (median 1.08). The PQ of the two *E. nuttallii* specimens measured in a continuous manner was calculated for the period of higher light intensities after 10 a.m. For the first *E. nuttallii* specimen the PQ was between 0.81 and 1.66 (median 1.05). The PQ of the second *E. nuttallii* specimen ranged between 0.47 and 0.79 (median 0.54), obviously because of lower oxygen evolution at a CO_2_ uptake comparable to the first specimen (c.f Figure 5B). The oxygen concentration did not exceed 120 μM during the *in situ* measurements in the pond and the carbon dioxide concentration was above 130 μM (see Supplementary Table 2). Even though photorespiration should not be enhanced at oxygen concentration below air saturation (Burris, 1981), these first results already emphasize the need for simultaneous assessment of oxygen and carbon turnover *in situ*.

### Future Work

The use of the presented, portable incubation chamber with an automated water exchange process enables prolonged *in situ* measurements.

Still, technical improvements shall be done in the future. The small spherical light sensor can be inserted into the incubation chamber instead of being attached to the side of the measurement device. Even though heat exchange with the surrounding water stabilized the temperature inside the device, an additional heat exchanger shall divert the heat energy from the peristaltic pump. The depth for *in situ* measurements is restricted by the length of the cable of the light sensor (2.8 m excluding the connective part) and of the polymeric optical fibers (5 m). This limitation could be resolved by supplying the sensors with an independent power supply and wireless data loggers as in Staudinger et al. (2018).

Currently, we see limitations in the calculation of the bicarbonate flux from changes in the pH and the carbon dioxide concentration. In some cases, it remains uncertain whether the change in the bicarbonate concentration results from active uptake or reflects a shift in the carbon species’ equilibrium. Additional measurements of the total alkalinity or the total inorganic carbon concentration should provide the basic information for this calculation. For plant species that can use bicarbonate, the carbon turnover should include the latter and not only carbon dioxide.

Ongoing environmental changes are requiring a comprehensive and accurate calculation of carbon balances in order to understand the consequential response, from individuals to the ecosystem level. The presented method shall improve insight into the metabolism of aquatic organisms as it is indispensable to consider both, the oxygen and carbon species turnover.

## Data Availability Statement

The raw data supporting the conclusions of this article will be made available by the authors, without undue reservation.

## Author Contributions

AL designed and built the underwater measurement device. NS and AS conducted the experiments in lab and field. AS analyzed the data and wrote the draft of the manuscript with contribution from MK. MK supervised the project and provided guidance in all steps. All authors read and approved the final version of the manuscript.

## Conflict of Interest

The authors declare that the research was conducted in the absence of any commercial or financial relationships that could be construed as a potential conflict of interest.

## Publisher’s Note

All claims expressed in this article are solely those of the authors and do not necessarily represent those of their affiliated organizations, or those of the publisher, the editors and the reviewers. Any product that may be evaluated in this article, or claim that may be made by its manufacturer, is not guaranteed or endorsed by the publisher.
