# Supplementary material for: Portable Measurement System for in situ Estimation of Oxygen and Carbon Fluxes of Submerged Plants
Source: Front Plant Sci. 2021 Nov 5;12:765089. doi: 10.3389/fpls.2021.765089 (PMC8604185; doi:10.3389/fpls.2021.765089)
Supplement: Supplementary file 1 [file Data_Sheet_1.docx]

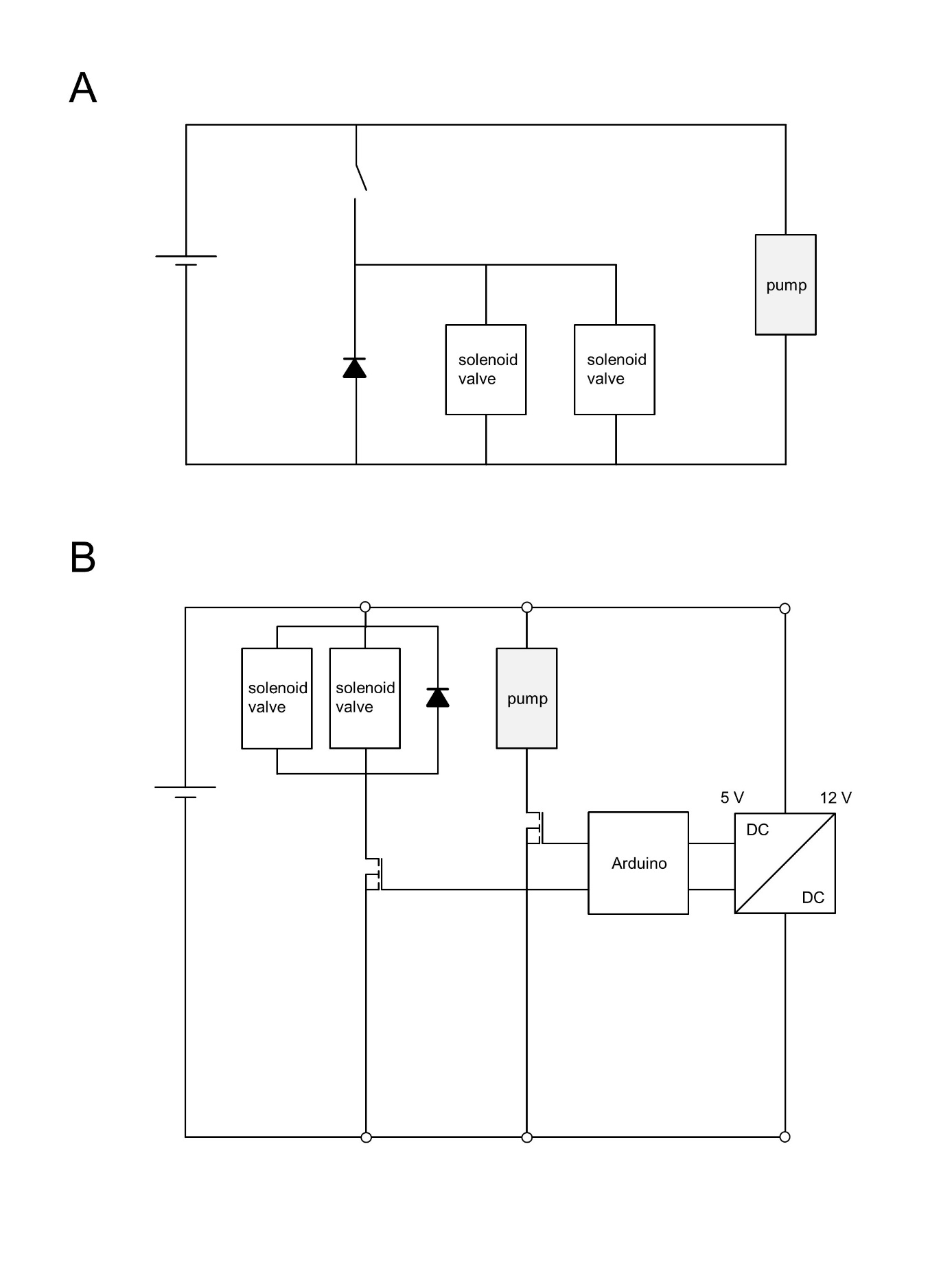


Figure 1. Circuit diagrams of the measurement device and the control unit. (A) Manual switching of the valves. (B) An Arduino microcontroller was implemented for the regulation of the pump and the valves. A routine was set with specific durations for the modes *flush* and *measurement*. As soon as the routine was started, the pump began to work.

Table 1: Characteristics of the used sensor foils. The pCO_2_ sensor was only available as prototype as the experiments were conducted.

|  | type | measurement range | resolution | accuracy | response time (t_90_) |
| --- | --- | --- | --- | --- | --- |
| Oxygen | SP-PSt3-NAU | 0 to 1400 µM | 1.4 µM at 283.1 µM  0.14 µM at 2.83 µM |  | < 40 s |
| pCO_2_ | CD2 | 100 to 10000 ppm * |  | 50 ppm * | 3 to 10 min *^/^** |
| pH | SP-HP5-SA | 5.5 to 8.5 | ± 0.01  (at pH 7) | ± 0.05  (at pH 7) | < 120 s |
| * preliminary, ** at T ≥ 20°C depending on pCO_2_ difference | | | | | |

Table 2: Date, time and environmental parameters at the beginning of each *in situ* measurement. Temperature in °C, oxygen concentration in µmol L^-1^, partial pressure of carbon dioxide in hPa.

| Specimen | Date | Time | Temperature | c(O_2_) | pCO_2_ | pH |
| --- | --- | --- | --- | --- | --- | --- |
| Elodea #1 | July 28th, 2020 | 06:42 | 20.6 | 4.6 | 5.5 | 7.21 |
|  |  | 07:12 | 20.6 | 7.1 | 5.2 | 7.21 |
|  |  | 07:41 | 20.6 | 9.8 | 5.3 | 7.22 |
|  |  | 08:11 | 20.5 | 9.5 | 5.4 | 7.21 |
|  |  | 08:40 | 20.5 | 13.4 | 5.2 | 7.21 |
|  |  | 09:10 | 20.6 | 9.9 | 5.3 | 7.23 |
|  |  | 09:40 | 20.6 | 9.5 | 4.9 | 7.22 |
|  |  | 10:09 | 20.7 | 16.2 | 4.8 | 7.22 |
|  |  | 10:54 | 21.2 | 28.0 | 4.3 | 7.19 |
|  |  | 11:23 | 21.1 | 39.4 | 4.2 | 7.23 |
|  |  | 11:53 | 21.2 | 48.7 | 4.1 | 7.22 |
|  |  | 12:22 | 21.4 | 60.2 | 3.7 | 7.23 |
|  |  | 12:52 | 21.6 | 73.1 | 3.6 | 7.25 |
|  |  | 13:22 | 21.9 | 89.7 | 3.5 | 7.25 |
|  |  | 13:51 | 22.2 | 105.1 | 3.8 | 7.23 |
| Elodea #2 | July 29th, 2020 | 06:16 | 19.7 | 0 | 5.6 | 7.16 |
|  |  | 06:45 | 19.6 | 0.4 | 5.5 | 7.17 |
|  |  | 07:15 | 19.4 | 2.3 | 5.4 | 7.17 |
|  |  | 07:44 | 19.3 | 4.7 | 5.4 | 7.17 |
|  |  | 08:14 | 19.3 | 5.1 | 5.5 | 7.18 |
|  |  | 08:39 | 20.0 | 7.8 | 5.5 | 7.15 |
|  |  | 09:08 | 19.3 | 12.0 | 5.5 | 7.16 |
|  |  | 09:38 | 19.4 | 27.1 | 5.6 | 7.16 |
|  |  | 10:08 | 19.4 | 70.6 | 5.1 | 7.17 |
|  |  | 10:42 | 19.8 | 49.1 | 5.6 | 7.17 |
|  |  | 11:11 | 19.9 | 46.6 | 5.5 | 7.18 |
|  |  | 12:29 | 20.1 | 36.5 | 6.1 | 7.14 |
|  |  | 12:59 | 20.3 | 37.5 | 6.4 | 7.12 |
|  |  | 13:28 | 20.6 | 43.9 | 6.1 | 7.13 |
|  |  | 13:58 | 20.9 | 68.6 | 5.8 | 7.14 |
| Ceratophyllum #1 | August 10th, 2020 | 10:04 | 20.4 | 82.3 | 6.4 | 7.21 |
| Ceratophyllum #2 |  | 10:45 | 20.4 | 42.9 | 6.9 | 7.15 |
| Ceratophyllum #3 |  | 11:13 | 20.7 | 93.0 | 6.4 | 7.16 |
| Ceratophyllum #4 |  | 11:40 | 20.9 | 112.0 | 5.8 | 7.16 |
| Ceratophyllum #5 |  | 12:13 | 21.1 | 111.2 | 5.9 | 7.13 |
| Ceratophyllum #6 |  | 12:40 | 21.3 | 120.4 | 5.9 | 7.14 |
